# Editorial: Clinical imaging, neurophysiological, neuropathology and neuroethics studies on disorders of consciousness, coma mechanisms, and theories of consciousness: a unifying attempt

**DOI:** 10.3389/fneur.2026.1803326

**Published:** 2026-03-02

**Authors:** Diego Iacono, Manuel Trachsel, Thanh G. Phan, Charlène Aubinet

**Affiliations:** 1 Neuropathology Research, Biomedical Research Institute of New Jersey (BRInj), Cedar Knolls, NJ, United States; 2 Neuroscience Research, MidAtlantic Neonatology Associates (MANA), Department of Pediatrics, Atlantic Health System (AHS), Morristown, NJ, United States; 3 Neurodevelopmental Research Lab, Biomedical Research Institute of New Jersey (BRInj), Cedar Knolls, NJ, United States; 4 Department of Neurology, Thomas Jefferson University, Philadelphia, PA, United States; 5 Clinical Ethics Unit, University Hospital Basel, University Psychiatric Clinics Basel, Geriatric University Hospital FELIX PLATTER Basel, University Children's Hospital Basel, Basel, Switzerland; 6 Faculty of Medicine, University of Basel, Basel, Switzerland; 7 Department of Medicine, School of Clinical Sciences at Monash Health, Monash University, Melbourne, VIC, Australia; 8 Department of Neurology, Monash Medical Centre, Melbourne, VIC, Australia; 9 Coma Science Group, GIGA-Consciousness, GIGA Institute, University of Liège, Liege, Belgium; 10 NeuroRehab and Consciousness Clinic, Neurology Department, University Hospital of Liège, Liege, Belgium; 11 Psychology and Neuroscience of Cognition Research Unit, University of Liège, Liege, Belgium

**Keywords:** coma, consciousness, disorders of consciousness (DOC), neuroethics, unifying approach

Understanding the nature of consciousness and alleviating the suffering of patients with Disorders of Consciousness (DoC) remains one of the most profound challenges in modern neuroscience and medicine. The transition from the acute phase of coma to Unresponsive Wakefulness Syndrome (UWS) or Minimally Conscious State (MCS) presents a complex landscape requiring the integration of theoretical physics, neuroanatomy, advanced diagnostics, therapeutic intervention, and rigorous ethical consideration. This Research Topic aims to bridge these distinct yet interconnected fields. The contributing articles offer a panoramic view of the current state of DoC research, moving from the theoretical underpinnings of conscious experience to the practicalities of neuromodulation and the ethical gravity of end-of-life decisions.

## Theoretical foundations and neuroanatomical substrates

The quest to investigate the physical substrate and mechanisms of consciousness continues to drive theoretical innovation, as well as philosophical and ethical discussions. In a novel theoretical contribution, Strupp introduces a variation of Electromagnetic Field Theory in the form of Electromagnetic Ion Field Theory (EIFT) of consciousness. Challenging standard synaptic-centric views, this theory postulates that phenomenal consciousness arises from the spatial integration of information within the brain's epineural electromagnetic field, specifically generated by ionic dynamics. Strupp proposes a shift from purely “*wired*” neuronal processing models to a “*field-based integration*,” suggesting experimental validations involving electromagnetic shielding to test these binding properties.

Grounding consciousness in neuroanatomy, Cacciatore et al. perform a systematic review to identify the thalamic nuclei most critical for consciousness. By synthesizing data from 167 studies, they identify the intralaminar nuclear group—specifically the centromedian-parafascicular complex (CM-Pf)—as the pivotal hub for generating and maintaining consciousness. Their findings reinforce the mesocircuit model and provide a strong anatomical rationale for selecting thalamic targets in neuromodulation therapies. Complementing this, Jung et al. investigated the structural integrity of the thalamo-dorsolateral prefrontal cortex tract (TDLPFCT) using diffusion tensor imaging (DTI). Their retrospective study reveals that the integrity of this tract, particularly in the less affected hemisphere, is significantly correlated with residual consciousness in prolonged DoC patients, highlighting the TDLPFCT as a potential biomarker for prognosis.

## Advances in neurophysiological diagnosis and prognosis

As theoretical understanding evolves, diagnostic tools must evolve too. Electroencephalography (EEG) remains a cornerstone of DoC assessment. Chen et al. provide a comprehensive bibliometric analysis of EEG application in DoC over the last two decades. Their review of 1,639 publications highlights the field's rapid expansion, identifying key research hotspots such as the differentiation between UWS and MCS). Building on this trend, Zhang et al. propose a multimodal approach to diagnosis. By combining resting-state (eyes closed) EEG with auditory-evoked potentials and utilizing machine learning (Support Vector Machine), they demonstrate that integrating non-linear dynamic features—such as spatiotemporal correlation entropy—significantly improves the accuracy of distinguishing MCS from UWS compared to unimodal assessments. Simultaneously, the prognostic value of EEG is scrutinized by Mori et al., who compare traditional visual EEG grading against quantitative EEG (qEEG) spectral analysis. Their retrospective study suggests that while visual interpretation remains robust, combining qEEG features (particularly central alpha power) with clinical factors like rehabilitation status significantly enhances the prediction of neurological recovery, advocating for a multimodal prognostic framework.

## Optimizing therapeutic interventions

The translation of diagnostic insight into therapeutic action is a critical theme of this Research Topic. Deep Brain Stimulation (DBS) and Spinal Cord Stimulation (SCS) are promising avenues for restoring consciousness, yet patient selection and technical precision are paramount. Raguž et al. address the challenge of DBS candidate selection by integrating qualitative and quantitative MRI analyses. Their study demonstrates that specific structural markers—such as striatal volume, thalamic atrophy, and leukoaraiosis—can accurately predict DBS candidacy, offering a standardized decision-support tool for centers lacking advanced functional imaging. In the realm of Spinal Cord Stimulation, He et al. investigate the mechanical complications of therapy. Their retrospective analysis reveals that electrode shift in the cervical region negatively correlates with patient outcomes. They identify anatomical factors, such as spinal canal diameter, that may influence stability, emphasizing that precise surgical placement is as critical as the stimulation parameters themselves.

## Ethical considerations in end-of-life care

Finally, the management of DoC is inextricably linked to profound ethical conflicts. Li et al. provide a crucial perspective from China, surveying healthcare providers on the Withdrawal of Life-Sustaining Treatment (WLST) and Advanced Directives (ADs). The study reveals a cultural hesitation toward WLST, with only a minority supporting the withdrawal of artificial nutrition or hydration. Almost half of assessed healthcare professionals had never heard of ADs before. Nevertheless, the study highlights an emerging support for ADs among professionals, suggesting a potential shift in the ethical landscape of end-of-life care in UWS patients in China.

Collectively, these articles illustrate that the future of DoC management lies in unification. We are moving toward a paradigm where theoretical physics inform neuroanatomy, which in turn guides targeted neuromodulation, all underpinned by precise, multimodal diagnostics and governed by evolving ethical frameworks. This Research Topic serves not only as a repository of current findings but as a roadmap for the interdisciplinary collaboration required to unlock the mysteries of consciousness and improve the lives of those disconnected from it.

